# A Simplified Herbal Formula Improves Cardiac Function and Reduces Inflammation in Mice Through the TLR-Mediated NF-κB Signaling Pathway

**DOI:** 10.3389/fphar.2022.865614

**Published:** 2022-06-06

**Authors:** Xiaoming Dong, Xiaowei Han, Xiaojiao Zhang, Sijing Li, Ziru Li, Jinhua Kang, Jialin Jiang, Shihao Ni, Lu Lu, Zhiling He, Haoming Huang, Shaoxiang Xian, Tianhui Yuan, Zhongqi Yang, Wenjie Long, Zemin Wan

**Affiliations:** ^1^ The First Clinical Medical College, Guangzhou University of Chinese Medicine, Guangzhou, China; ^2^ Lingnan Medical Research Center, Guangzhou University of Chinese Medicine, Guangzhou, China; ^3^ The Second Clinical Medical College, Guangzhou University of Chinese Medicine, Guangzhou, China; ^4^ Department of Cardiology, The Second Affiliated Hospital of Guangzhou University of Chinese Medicine, Guangzhou, China; ^5^ Department of Radiology, The First Affiliated Hospital of Guangzhou University of Chinese Medicine, Guangzhou, China; ^6^ Department of Geriatrics, The First Affiliated Hospital of Guangzhou University of Chinese Medicine, Guangzhou, China; ^7^ Department of Laboratory Medicine, The Second Affiliated Hospital of Guangzhou University of Chinese Medicine, Guangzhou, China

**Keywords:** chronic heart failure, inflammation, Chinese medicine, Nuanxinkang tablet, TLR-mediated NF-κB signaling pathway

## Abstract

Nuanxinkang tablet (NXK), a Chinese herbal formula, can improve heart function and quality of life in patients with chronic heart failure (CHF). However, the mechanisms of action of NXK are not fully understood. In this study, we investigated the effects of NXK on inflammation in the CHF mouse model. This model was established by transverse aortic constriction (TAC) and treated with NXK for 8 weeks. Then, the cardiac function and myocardial fibrosis were evaluated. The monocytes/macrophages were evaluated by immunofluorescence. The mRNA levels of *IL-1*β, *IL-6*, *TNF-*α, *ICAM-1*, and *VCAM-1* were measured by quantitative real-time polymerase chain reaction (qRT-PCR), while TLR4, MyD88, NF-κB p65, P-IκBα, TLR2, TLR7 and TLR9 protein levels were evaluated by Western blot. The results showed that NXK improved the left ventricular ejection fraction (LVEF) and left ventricular end-systolic dimension, reversed myocardial fibrosis, and inhibited pro-inflammatory (CD11b + Ly6C+) monocytes/macrophages in the TAC mouse model. NXK also reduced the mRNA and protein levels of the above markers. Taken together, NXK improved heart function and reduced inflammation through the TLR-mediated NF-κB signaling pathway, suggesting that it might be used as an innovative treatment strategy for CHF.

## Introduction

Chronic heart failure (CHF) is one of the leading causes of high morbidity and mortality worldwide. Over the last 2 decades, the clinical use of neurohormonal and sympathetic nervous system blockers, including the combination of angiotensin-converting enzyme inhibitor (ACEI)/angiotensin II receptor blockers (ARBs), β-blockers, and vasodilators, has revolutionized the care of patients with CHF (Packer et al., 2021). However, these drugs are not fully efficacious against CHF (Webber et al., 2020), and it has been reported that 2%–17% of CHF patients die at the first hospital admission. Ponikowski et al. (2014) reported a mortality rate of 17%–45% within 1 year after admission and >50% within 5 years. Therefore, developing new, safe, and effective therapeutics for CHF is crucial.

CHF involves a complex pathological process that simultaneously interferes with multiple genes and signaling pathways (Chen et al., 2020). Cardiac inflammation is an important pathogenesis of CHF and leads to adverse clinical outcomes. Necrotic, stressed and damaged heart cells can release dangerous substances as signals, which are called danger-associated molecular patterns (DAMPs). Several DAMPs can activate toll-like receptors (TLRs) and NF-κB signaling pathways, which would stimulate the expression of pro-inflammatory cytokines such as TNF-α, IL-1β, IL-18, etc (Prabhu and Frangogiannis, 2016).

Multi-targeted drugs for CHF treatment, such as traditional Chinese medicine (TCM), have attracted increasing attention in previous clinical trials (Xian et al., 2015). Yu et al. (2019) conducted a retrospective analysis of TCM in a random sample of hospitalizations for heart failure within a random sample of Western medicine hospitals in China in 2015 and found a similar effect of the two drugs for treating CHF.

Nuanxinkang (NXK) is a Chinese herbal formula that is widely utilized for CHF treatment in China. It is composed of *Radix ginseng Rubra* (Hong Shen) and *llex pubescens* (Mao DongQing). Our preclinical study showed that NXK has an anti-apoptotic effect against CHF, which is mediated by the AKT signaling pathway (Chen et al., 2019). Another study showed that combination therapy with NXK and basic CHF treatment effectively improved cardiac function in CHF patients compared to basic CHF treatment alone. It also exhibited better therapeutic effects on improving quality of life, controlling inflammation, and regulating neurohormones (Zhang, 2012). However, the exact mechanisms underlying the effects of NXK remain unclear.

In this study, we investigated the pharmacological functions of NXK on CHF mouse model established through transverse aortic constriction (TAC). We found that NXK is effective against CHF by down-regulating the signaling pathway of inflammation. This study provided evidence for an innovative treatment strategy and potential therapeutic targets for CHF.

## Materials and Methods

### NXK Preparation

NXK consists of two herbs, *Radix ginseng Rubra* and *llex pubescens*. The herbs were mixed according to the ratio of 23:77. The concentrated NXK powders were produced by Sci-tech Industrial Park, Guangzhou University of Chinese Medicine, under internationally certified good manufacturing practice guidelines (Serial Number: TA2017014).

### Animal Model

Four-week-old C57BL/6 male mice were obtained from the Guangdong Animal Experimental Center. The mice were housed in an environment with a temperature of 22 ± 1°C, relative humidity of 50 ± 1%, and a light/dark cycle of 12/12 h. The animal experiment protocols conformed to the Guidelines for the Care and Use of Laboratory Animals published by the National Academy Press (National Institutes of Health Publication No. 85-23, revised 1996) and were approved by the Animal Care and Use Committee of Guangzhou University of Chinese Medicine (permit number: 20200608003).

The mice were divided into two groups: the TAC group and the sham group. Heart failure was achieved *via* transverse aortic constriction (TAC) on eight-week-old mice. The animals were intubated and ventilated with room air using a small-animal respirator (Harvard Inspira ASVv, Massachusetts, United States) and anesthetized with 1.0% isoflurane inhalation. The left side of the chest was then opened at the second intercostal area. A 7-0 silk string was passed under the transverse thoracic aorta between the innominate and left common carotid arteries and snared around the aorta with a 28-gauge needle. After ligation, the needle was removed, and the skin was sutured. The sham group underwent the same surgery but without constriction.

The mice were then divided into six groups (10 mice/group): Sham, TAC, TAC + low dose NXK (NXK-L), TAC + medium-dose NXK (NXK-M), TAC + high-dose NXK (NXK-H), and TAC + Perindopril. Intramuscular injection of penicillin was administered to all groups for 3 days.

### Drug Administration

To determine the optimal dose of NXK, we used 0.42 g/kg/d as the low dose, 0.83 g/kg/d as the medium dose, and 1.65 g/kg/d as the high dose. Three days after surgery, the TAC + NXK-L, TAC + NXK-M, and TAC + NXK-H groups were intragastrically administered 0.42 g/kg/d, 0.83 g/kg/d, and 1.65 g/kg/d NXK, while the TAC + Perindopril group was given 3.6 g/kg for 28 days.

Mice in the lipopolysaccharide (LPS) and TAK-242 groups were intraperitoneal injected with saline or TLR4 inhibitor (TAK-242; 2 mg/kg), respectively, followed by LPS (12 mg/kg; intraperitoneal injection) 1 h after the surgery. The sham and TAC groups were given sterile water. The mice were used for experiments 8 weeks after the surgery.

### Echocardiography

Echocardiography studies were performed using a Vevo 2,100 imaging system (FUJIFILM Visual Sonics Inc., Canada). The mice were anesthetized with 2% isoflurane inhalation. The M-mode image of the middle layer of the nipple muscle was used to measure the left ventricular end-systolic volume (LVESV), left ventricular end-diastolic volume (LVEDV), left ventricular end-diastolic diameter (LVEDD), left ventricular end-systolic diameter (LVESD), end-systolic volume (ESV), end-diastolic volume (EDV), left ventricular posterior wall end-diastole (LVPWD) and end-systole (LVPWS). The left ventricular ejection fraction (LVEF) was calculated as (LVEDV—LVESV)/LVEDV; the left ventricular fraction shortening (LVFS) was calculated as (LVEDD—LVESD)/LVEDD; the stroke volume (SV) was calculated as (EDV − ESV). Blind observers used Vevo 2,100 workstation 1.7.1 to analyze the digital images offline.

### Masson’s Trichrome Staining and Immunofluorescence

The heart tissue was fixed in 4% paraformaldehyde and then immersed in graded series of alcohol: 50% ethanol for 30 min, 70% ethanol for 60 min, 80% ethanol for 60 min, 90% ethanol twice for 30 min each, and absolute ethanol for 30 min, followed by 60 min in isobutanol + carbonic acid. Subsequently, the specimens were embedded in wax for 90 min and cooled. Masson’s Trichrome staining was performed on the paraffin-embedded heart sections to assess myocardial fibrosis. Sections were also blocked and then incubated with antibodies against CD11b and Ly6C. Nuclei were stained with DAPI. Nikon Eclipse microscope with NIS Elements software was used to capture the images. ImageJ software was used to measure the area of cardiac fibrosis (the ratio of fibrotic area to total myocardial area). At least 20 fields (200X) were analyzed in each section.

### Quantitative Real-Time PCR Analysis

The heart samples were snap-frozen in liquid nitrogen and placed on ice for 30 min before extracting total RNA using TRIzol reagent. cDNA was synthesized using TIANScript RT kit^®^ (TIANGEN BIOTECH, China) according to the manufacturer’s instructions. The RT-PCR amplification reaction was 20 μl, containing 10 μl LightCycler FastStart DNA MasterMix, 1 μl cDNA, 7 μl RNase/DNase-free water, and 500 nM of each primer. The primer sequences are listed in Supplementary Table S1. The expression of the target gene was quantified by the standard curve and the 2-ΔΔCt method. β-actin was used as an internal control to normalize the levels of the target genes.

### Western Blotting

Total protein was extracted from the heart tissue according to the instructions provided in the total protein extraction kit, and the protein concentration was determined (Ca. P0013B, Beyotime, Shanghai, China). The proteins were separated by sodium dodecyl sulfate-polyacrylamide gel electrophoresis (SDS-PAGE) (10% and 12%). Then, the protein was transferred to polyvinylidene fluoride (PVDF) membrane (Ca. IPVH00010, EMD Millipore, Billerica, MA, United States), blocked with 5% skimmed milk in Tris-buffered saline-Tween 20 (TBST) for 90 min at 37°C, and probed with the following primary antibodies: TLR4 at 1:500 dilution; MyD88, NF-κB p65, P-IκBα, IκBα, TLR2, TLR7, TLR9 and beta-tubulin at 1:1,000 dilution. Subsequently, the membranes were incubated with the corresponding secondary antibody. The immunoreactive bands were developed using ECL, and the ProteinSimple imaging system was utilized to scan the target protein. ImageJ software was used to analyze the image.

### Statistical Analysis

All values were reported as mean ± standard error of the mean (SEM). The normality of data distribution was evaluated using the Shapiro-Wilk test. Data with skewed distribution were transformed by calculating their natural logarithm to approximate normal distribution. One-way analysis of variance (ANOVA) followed by Dunnett’s test or the Student Newman-Keuls (SNK) test were used to analyze the differences between the groups. Differences were considered statistically significant at *p* < 0.05.

## Results

### NXK Improved the Cardiac Function of CHF

**FIGURE 1:**
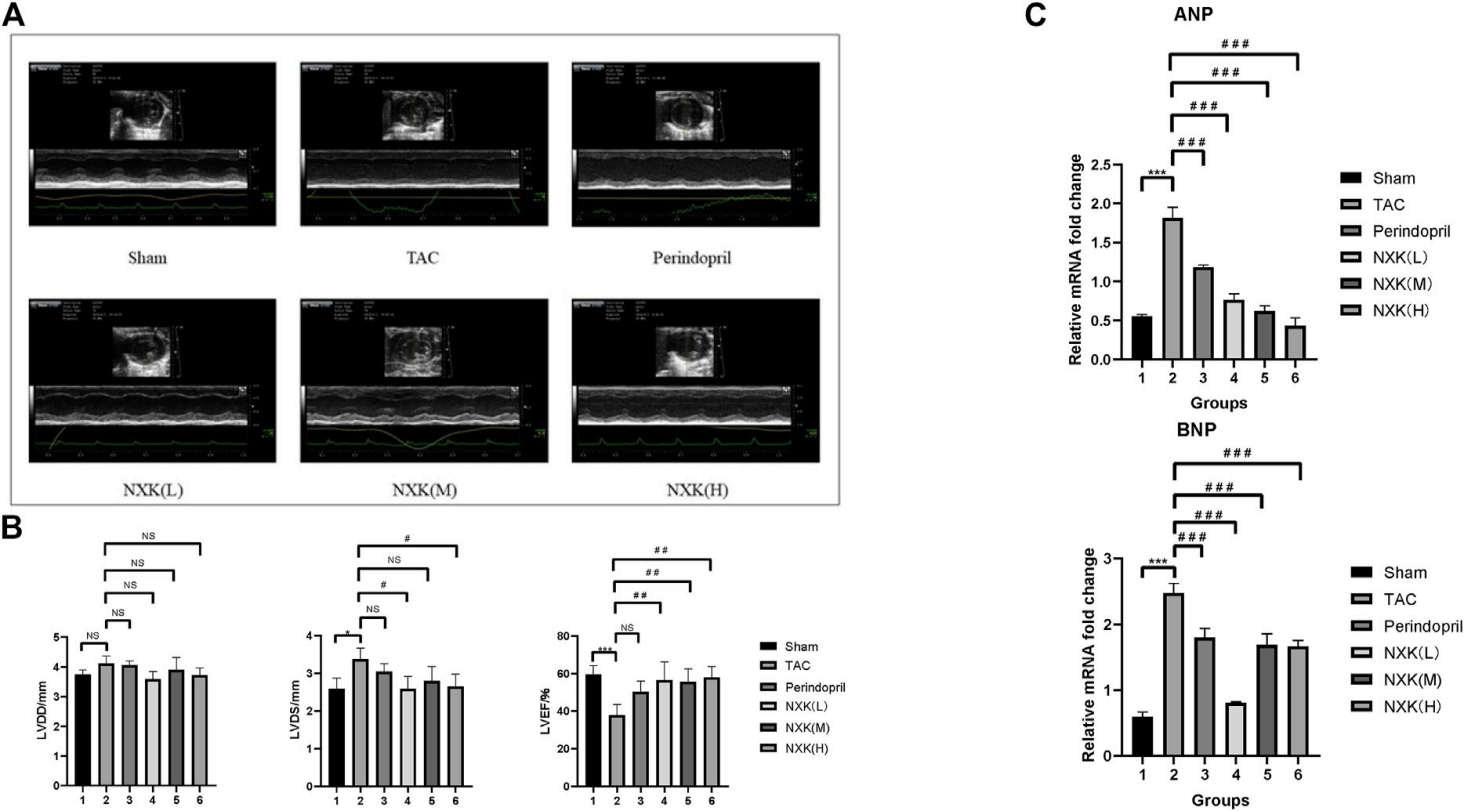
NXK improves the cardiac function of mice with heart failure. **(A)** Representative M-mode images via echocardiography. **(B)** Representative cardiac function (*n* = 10 per group). LVDD (mm), left ventricular end-diastolic dimension; LVDS (mm), left ventricular end-systolic dimension; LVEF (%), left ventricular ejection fraction. **(C)** mRNA expression levels of ANP and BNP were detected using RT-PCR. Quantification was performed after normalization to β-actin (*n* = 10 per group). Values are shown as means ± SD, <0.05, compared to the sham group *p < 0.05, **p < 0.01, ***p < 0.001, compared to the Tac group #*p* < 0.05, ##*p* < 0.01, ###*p* < 0.001.

Cardiac function was evaluated *via* echocardiography (Figure 1A; Table 1). Compared to the sham group, LVDS was increased in the TAC group (*p* < 0.05), while LVEF and LVFS were decreased (all *p* < 0.01). Compared to the TAC group, the three NXK groups showed significant increases in LVEF and LVFS (all *p* < 0.01, Figure 1B; Table 1), thus suggesting a protective role of NXK in cardiac hypertrophy. However, there were no differences in LVPWD and LVPWS between the groups (all *p* > 0.05).

**TABLE 1:** Cardiac function of each group (mean ± SD).

Group	Sham	TAC	Perindopril	NXK (L)	NXK (M)	NXK (H)
LVDD (mm)	3.75 (0.15)	4.12 (0.26)	4.06 (0.14)	3.72 (0.25)	3.91 (0.40)	3.59 (0.26)
LVDS (mm)	2.59 (0.28)	3.37* (0.29)	3.04 (0.22)	2.65^#^ (0.33)	2.80 (0.38)	2.59^#^ (0.32)
LVEDV (μL)	60.13 (5.54)	75.34 (10.79)	72.59 (6.09)	59.33 (9.56)	67.28 (16.73)	54.37 (8.95)
LVFS (%)	31.05 (3.24)	18.28** (3.13)	24.94 (3.44)	29.77^##^ (3.85)	29.24^##^ (3.63)	29.25^##^ (6.40)
LV Mass (mg)	84.98 (5.69)	112.90 (24.57)	97.48 (6.58)	86.42 (12.27)	107.11 (18.95)	82.64 (5.40)
LVSV (μL)	35.34 (2.98)	28.60 (4.59)	36.22 (3.23)	33.04 (1.81)	37.08 (6.93)	29.58 (6.46)
CO (ml/min)	16.93 (2.72)	15.52 (3.31)	16.59 (2.16)	16.68 (1.60)	17.56 (3.76)	15.53 (3.44)
LVEF (%)	59.49 (4.84)	37.79*** (5.65)	51.55 (4.38)	57.97^##^ (5.82)	55.66^##^ (6.83)	57.73^##^ (8.33)
LVPWD (mm)	0.70 (0.13)	0.80 (0.06)	0.77 (0.05)	0.75 (0.18)	0.72 (0.12)	0.73 (0.14)
LVPWS (mm)	1.11 (0.17)	1.10 (0.14)	1.05 (0.09)	1.08 (0.17)	1.02 (0.16)	1.20 (0.12)

LVDD (mm), left ventricular end diastolic dimension; LVDS (mm), left ventricular end systolic dimension; LVEDV (μl), left ventricular end diastolic volume; LVFS (%), left ventricular left shortening fraction; LV MASS (mg) left ventricular myocardial weight; LVSV (μl), left ventricular end systolic volume; CO (ml/min), cardiac output; LVEF (%), left ventricular ejection fraction; LVPWD (mm), left ventricular posterior wall end diastole; LVPWS (mm), left ventricular posterior wall end systole. Compared to the sham group *p < 0.05, **p < 0.01, ***p < 0.001. Compared to the Tac group #*p* < 0.05, ##*p* < 0.01, ###*p* < 0.001.

RT-PCR was used to detect the RNA expression levels of BNP and ANP in all groups. The expression levels of BNP and ANP were significantly increased in the TAC group compared to the sham group (*p* < 0.001, Figure 1C, Supplementary Table S2). Furthermore, the expression levels of BNP and ANP in the Perindopril and the NXK low-, medium-, and high-dose groups were significantly lower than that of the TAC group (all *p* < 0.001, Figure 1C, Supplementary Table S2). Taken together, these results demonstrated the therapeutic efficacy of NXK in the heart failure model.

### NXK Improved Myocardial Fibrosis

**FIGURE 2:**
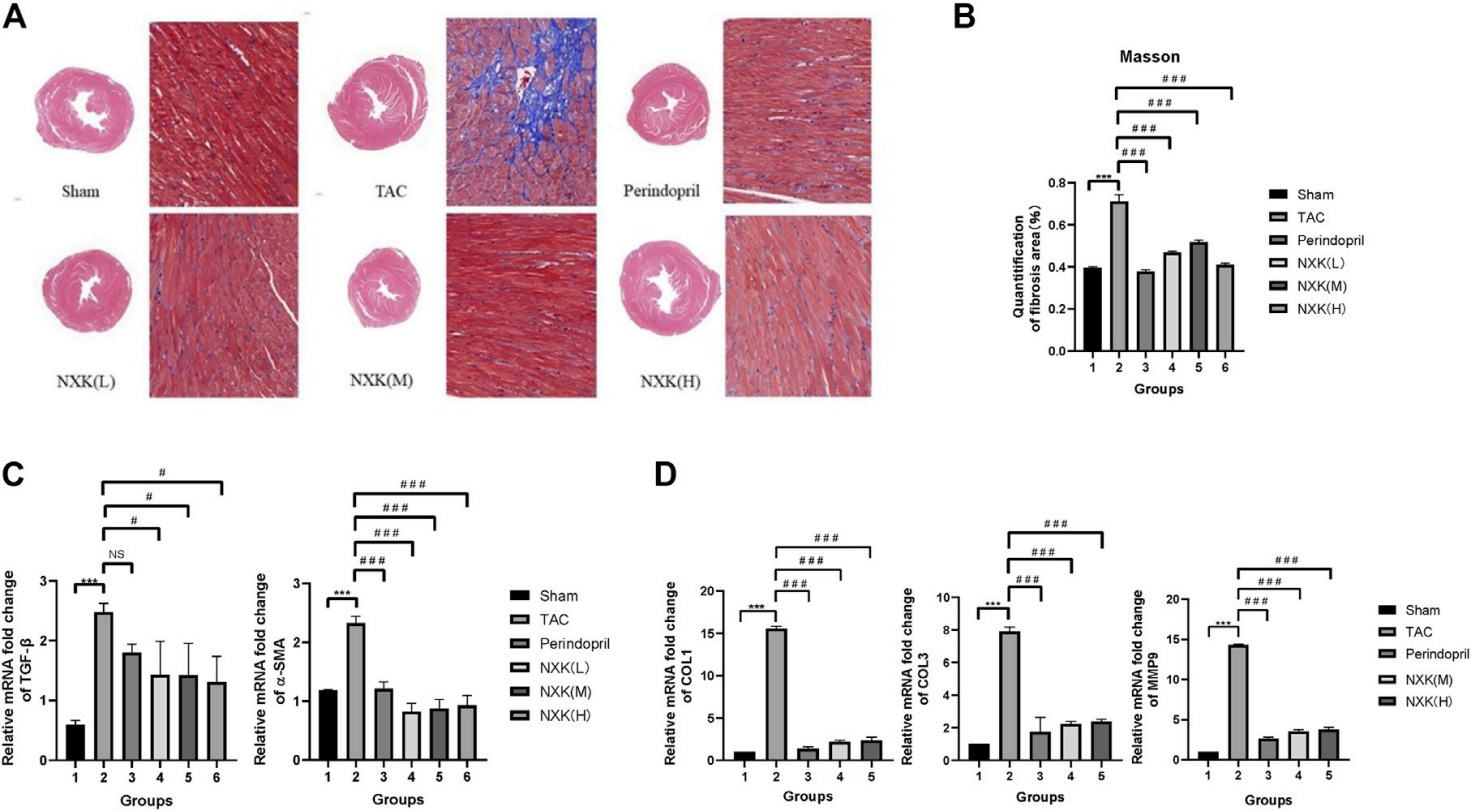
The effect of NXK on myocardial fibrosis in mice with heart failure. **(A)** Myocardial fibrosis was evaluated through Masson’s Trichrome staining (*n* = 10 per group). The fibrotic area is shown in blue. **(B)** Quantification of fibrosis area in the sham, TAC, low-, mid- and high-dose NXK groups. **(C)** mRNA expression levels of α-SMA and TGF-β quantified by RT-PCR (*n* = 10 per group). Quantification was performed after normalization to β-actin. **(D)** mRNA expression levels of Col1, Col3, and MMP9 quantified by RT-PCR (*n* = 6). Quantification was performed after normalization to GAPDH. Values are shown as means ± SD, compared to the sham group *p < 0.05, **p < 0.01, ***p < 0.001, compared to the Tac group #*p* < 0.05, ##*p* < 0.01, ###*p* < 0.001.

To further investigate the cardio-protective role of NXK, we examined the effect of NXK on myocardial fibrosis. HE staining showed that the hearts in the TAC group were significantly larger than that of the sham and drug groups, resulting in increased pressure load and myocardial hypertrophy. Next, we used Masson’s Trichrome staining to measure myocardial fibrosis. The degree of fibrosis was higher in the TAC group than in the sham and the drug groups (Figure 2A). Moreover, a larger fibrotic area was detected in the myocardium of the TAC group, where the greatest pressure load was observed compared to the sham group. In contrast, there was significantly smaller fibrotic area in the NXK group (Figures 2A,B, and Supplementary Table S3).

The mRNA expression levels of α-SMA, TGF-β, collagen I (Col1), collagen III (Col3), and matrix metalloprotein 9 (MMP9) were increased in the TAC group compared with the sham group (Figures 2C,D), and the mRNA expression levels of TGF-β in the NXK low, middle, high dose groups were significantly decreased vs. the TAC group (*p* < 0.05 vs. TAC, Figures 2C,D, Supplementary Table S4). The relative expression levels of Col1, Col3, α-SMA and MMP9 were significantly decreased in the NXK low, middle, high dose groups (*p* < 0.001 vs. TAC, Figures 2C,D, Supplementary Table S4). These results indicated that NXK treatment remarkably decreased myocardial fibrosis in the CHF model.

### NXK Reduced the Inflammation of CHF the Infiltration of Pro-Inflammatory Monocyte/Macrophages

**FIGURE 3:**
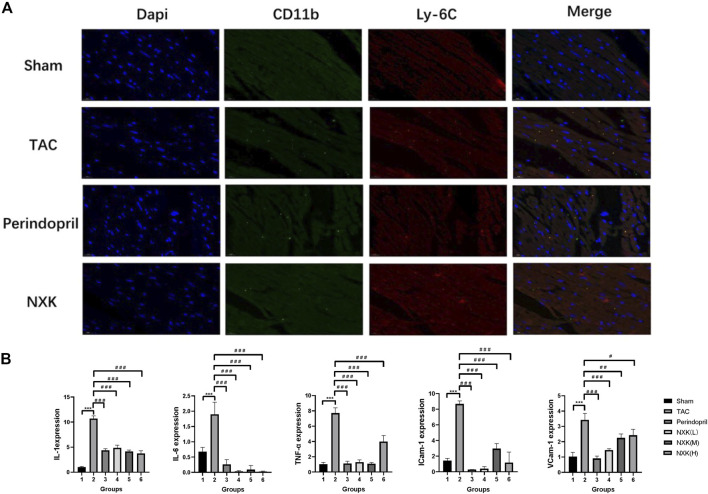
NXK reduces the inflammation due to heart failure by inhibiting the infiltration of pro-inflammatory monocytes/macrophages. **(A)** Representative images of heart tissue from the sham, TAC, perindopril, and NXK groups. DAPI staining (blue), CD11b (green), and Ly6c (red) are indicated (*n* = 6 per group). **(B)** mRNA expression levels of IL-1, IL-6, TNF-α, ICAM-1, and VCAM-1 quantified by RT-PCR (*n* = 10 per group). Values are shown as means ± SD, compared to the sham group *p < 0.05, **p < 0.01, ***p < 0.001, compared to the Tac group #*p* < 0.05, ##*p* < 0.01, ###*p* < 0.001.

Immunofluorescence demonstrated that CD11b + Ly6C + monocytes/macrophages were significantly increased in the model group. In contrast, infiltration of CD11b + Ly6C + monocytes/macrophages were inhibited in the Perindopril and NXK groups (Figure 3A).

Next, we detected the RNA expression levels of TNF-α, IL-6, IL-1, VCAM-1, and ICAM-1 among the groups. Compared to the sham group, the expression levels of IL-1, IL-6, TNF-α, VCAM-1, and ICAM-1 were significantly increased in the TAC groups (*p* < 0.001, Figure 3B; Table 2). Moreover, the expression levels of IL-1, IL-6, and TNF-α were decreased in the Perindopril group and the NXK high-, middle-, and low-dose groups (*p* < 0.001 vs. TAC). The results also showed decreased expression levels of VCAM-1 and ICAM-1 in the drug intervention group compared with the TAC group (*p* < 0.05, Figure 3B; Table 2).

**TABLE 2:** RNA expression levels of IL-1, IL-6, TNF-α, VCAM-1, and ICAM-1 (mean ± SD).

Group	Sham	TAC	Perindopril	NXK(L)	NXK(M)	NXK(H)
IL-1 expression	1.01 (0.14)	10.69^***^ (0.55)	4.36^###^ (0.32)	4.85^###^ (0.53)	4.12^###^ (0.28)	3.72^###^ (0.54)
IL-6 expression	1.20 (0.75)	1.38^***^ (0.77)	0.26^###^ (0.12)	0.03^###^ (0.02)	0.09^###^ (0.11)	0.02^###^ (0.22)
TNF-α expression	1.02 (0.21)	7.69^***^ (0.56)	1.11^###^ (0.24)	1.27^###^ (0.25)	1.09^###^ (0.13)	3.96^###^ (0.65)
VCam-1 expression	1.02 (0.23)	3.42^***^ (0.36)	0.90^###^ (0.13)	1.45^###^ (0.08)	2.25^##^ (0.22)	2.42^#^ (0.32)
ICam-1 expression	1.43 (0.23)	8.66^***^ (0.34)	0.27^###^ (0.01)	0.39^###^ (0.18)	2.94^###^ (0.54)	1.16^###^ (0.29)

Quantification was performed after normalization to β-actin. Compared to the sham group *p < 0.05, **p < 0.01, ***p < 0.001. Compared to the Tac group #*p* < 0.05, ##*p* < 0.01, ###*p* < 0.001.

### The Effect of NXK on the TLR4/NF-κB Signaling Pathway

**FIGURE 4:**
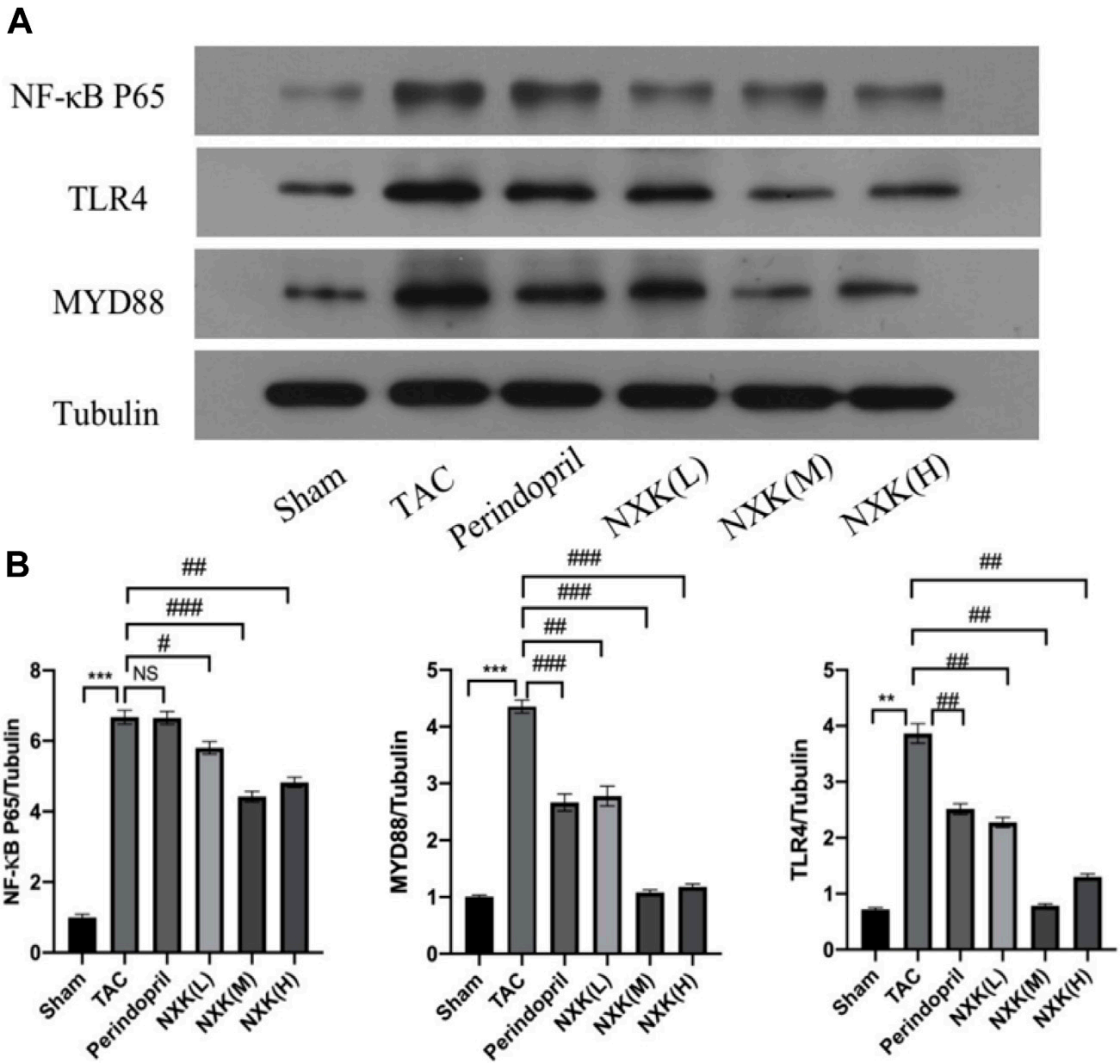
Western blot analysis was used to measure the protein level of TLR4/NF-κB pathway proteins. **(A)** Expression of TLR4, MyD88, NF-κB p65 proteins in different mice groups (*n* = 10 per group). **(B)** Quantification was performed after normalization to Tubulin. Values are shown as means ± SD, <0.05, compared to the sham group *p < 0.05, **p < 0.01, ***p < 0.001, compared to the Tac group #*p* < 0.05, ##*p* < 0.01, ###*p* < 0.001.

**FIGURE 5:**
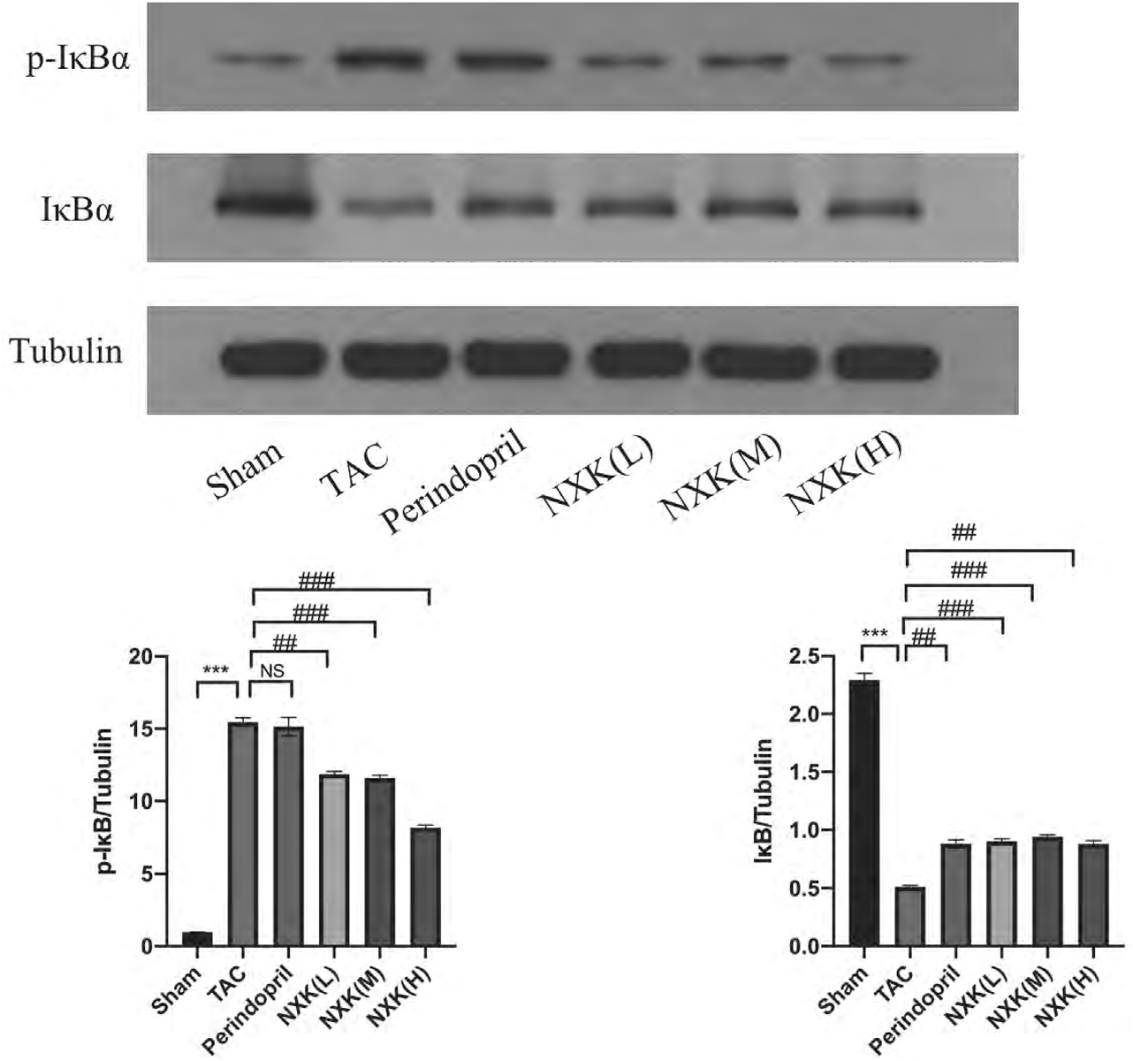
Western blot analysis was used to measure the protein levels of IκBα and P-IκBα. Expression levels of IκBα and P-IκBα proteins in different mice groups (*n* = 6 per group). Quantification was performed after normalization to Tubulin. Values are shown as means ± SD, compared to the sham group *p < 0.05, **p < 0.01, ***p < 0.001, compared to the Tac group #*p* < 0.05, ##*p* < 0.01, ###*p* < 0.001.

NF-κB is a pivotal transcription factor that regulates inflammation, immune responses, and cell survival. In this study, the levels of the target proteins, TLR4, MyD88, and NF-κB p65 **(** Figure 4 **)**, were significantly increased in the TAC group compared to the sham group. Moreover, the relative expression levels of TLR4 and MyD88 proteins in the high-, medium-, and low-dose groups of NXK and the Perindopril group were significantly lower compared to the TAC group (*p* < 0.01 vs. TAC, Figure 4 and Supplementary Table S5). The relative expression of NF-κB p65 was significantly lower in the high-, medium-, and low-dose groups compared to the TAC group (*p* < 0.05, Figure 4 and Supplementary Table S5). Besides, the expression of total IκBα and the phosphorylation of IκBα (P-IκBα) were further measured. The P-IκBα protein level was increased after TAC treatment and decreased after NXK treatment. Moreover, total IkBα was increased with decrease in p-IkBα after NXK treatment (*p* < 0.01 vs. TAC, Figure 5 and Supplementary Table S6).

**FIGURE 6:**
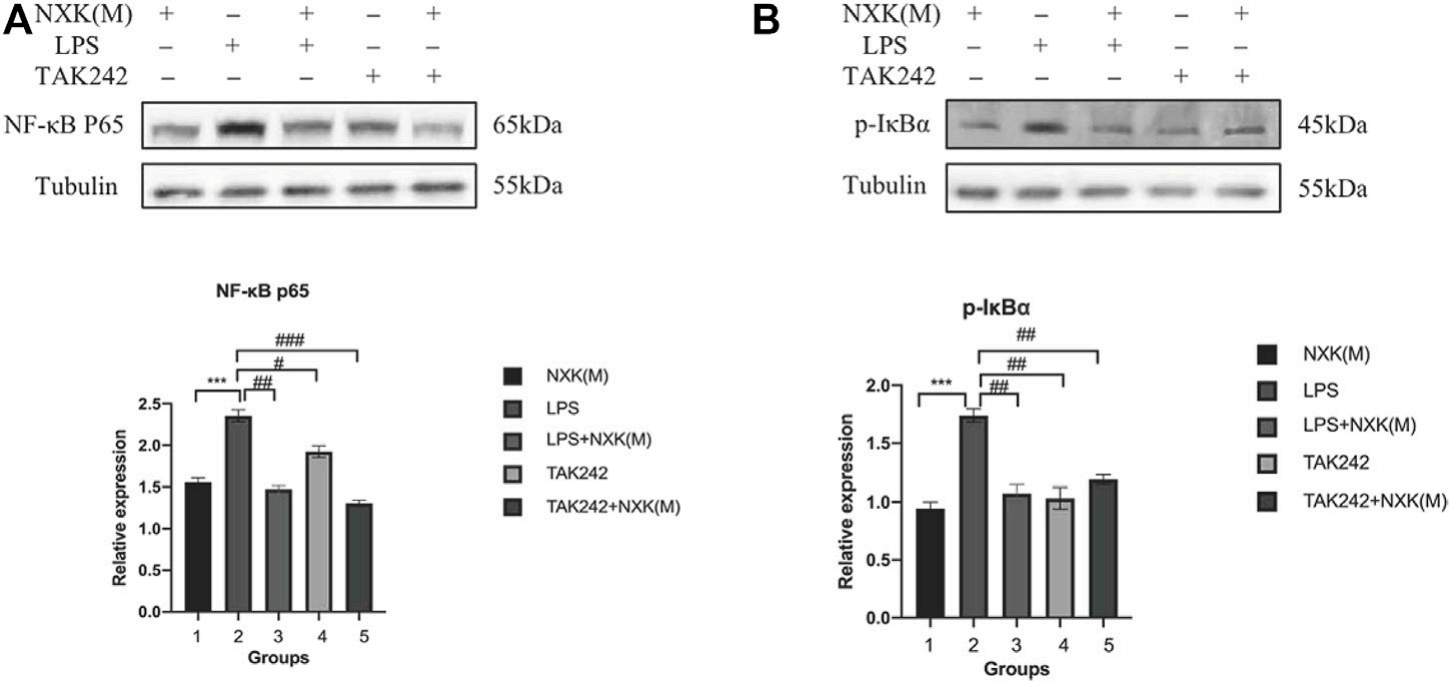
Western blot analysis was used to measure the protein levels of NF-κB p65 and P-IκBα, treated by NXK (M), TAK242, and LPS. **(A)** Expression of NF-κB p65 protein in different mice groups. **(B)** Expression levels of NF-κB p65 and P-IκBα proteins in different groups (*n* = 6 per group). Quantification was performed after normalization to Tubulin. Values are shown as means ± SD, *p < 0.05, **p < 0.01, ***p < 0.001, vs. LPS group.

Next, we used the TLR4 inhibitor TAK242 and agonist LPS to evaluate the effect of NXK. As shown in Figure 6A, the relative protein expression levels of NF-κB p65 were significantly decreased in the TAK242, NXK (M), LPS + NXK(M) and the TAK242 + NXK (M) groups compared with the LPS groups (*p* < 0.05, Figure 6A, Supplementary Table S7). Treatment with TAK242, NXK (M), LPS + NXK(M) and the TAK242 + NXK (M) significantly reduced the protein levels of P-IκBα (*p* < 0.01, Figure 6B, Supplementary Table S7).

**FIGURE 7:**
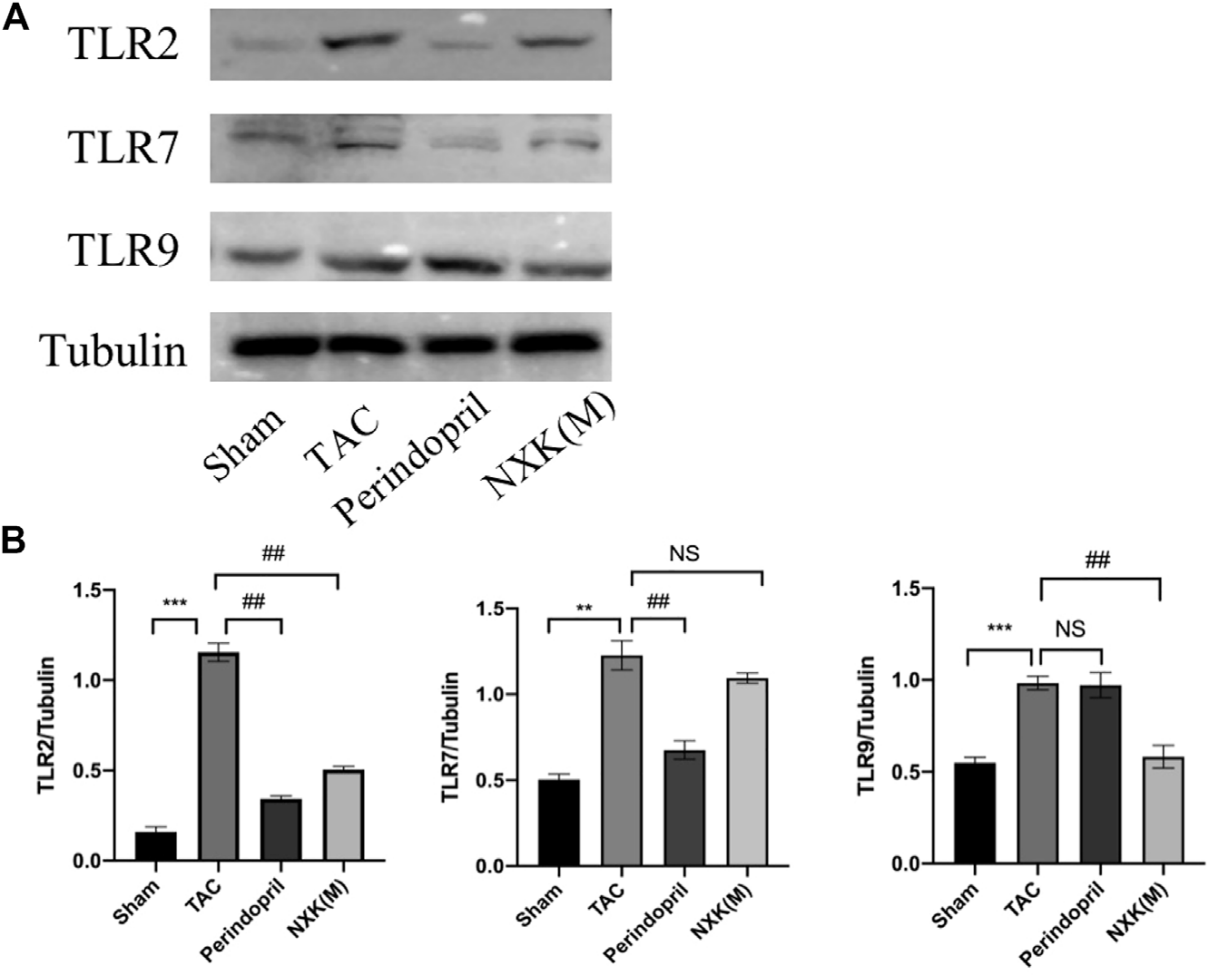
Western blot analysis was used to measure the protein levels of TLR2, TLR7, and TLR9 pathway proteins. **(A)** Expression levels of TLR2, TLR7, and TLR9 proteins in different mice groups and quantification was performed after normalization to GAPDH (*n* = 6 per group). **(B)** Values are shown as means ± SD, compared to the sham group *p < 0.05, **p < 0.01, ***p < 0.001, compared to the Tac group #*p* < 0.05, ##*p* < 0.01, ###*p* < 0.001.

The relative expression levels of TLR2, TLR7 and TLR9 were also evaluated. As shown in Figure 7A, the relative protein expression levels of TLR2, TLR7 and TLR9 were significantly increased in the TAC group compared with the sham group, and the protein levels of TLR2 and TLR9 in the NXK medium-dose group were significantly decreased compared with the TAC group (*p* < 0.01, Figure 7B). In addition, the expression of TLR7 in the NXK group showed no significant difference compared with the TAC group (*p* > 0.05, Figure 7B, Supplementary Table S8).

## Discussion

CHF is the final stage of all cardiovascular diseases (CVDs) (Kurlianskaya et al., 2020), and a common and potentially fatal disease. The signs and symptoms of CHF include shortness of breath, excessive fatigue, swelling of the legs (Tendera, 2005), and decreased exercise tolerance, which reduce the ability to engage in normal daily life activities (Long et al., 2020b). In most industrialized countries, the number of CHF patients has been rapidly increasing due to population aging and longer survival after acute myocardial infarction (McMurray and Stewart, 2002). Over the years, TCM has been used to supplement the treatment of CVD (Long et al., 2020a; Zhi et al., 2020). Previous studies have shown that NXK can improve cardiac function and inflammatory state of CHF patients (Zhang, 2012). However, the mechanism of action of NXK in the treatment of CHF remains unclear.

The present study suggested that a simplified herbal formula, NXK, can improve the LVEF and LVED, and reverse myocardial fibrosis. Moreover, NXK reduced inflammatory cytokines and inhibited pro-inflammatory monocytes/macrophages in the CHF mouse model. The results also indicated that NXK might inhibit CHF and anti-inflammatory activity through the TLR4/NF-κB signaling pathway, which is a classical signaling pathway of inflammation. Taken together, NXK was identified as an innovative treatment strategy for CHF.

CHF and inflammation interact in a vicious feedback loop (Pugliese et al., 2020). In terms of pathogenesis, the accumulation of inflammatory factors could lead to CHF-related complications, such as diabetes, obesity, and atherosclerosis (Tall and Yvan-Charvet, 2015). Inflammatory factors can also induce cardiac remodeling and cause cardiac mechanical motor dysfunction. Moreover, CHF induces and worsens the occurrence and progression of chronic inflammation (Jahng et al., 2016). Hence, inflammation has an important role in the occurrence, development, and prognosis of CHF, which includes inflammatory immune cell infiltration. The recruitment of T cells, pro-inflammatory monocytes and macrophages to the heart play a key role in CHF. This study found that NXK can improve inflammatory immune cell infiltration and inhibit pro-inflammatory (CD11b + Ly6C+) monocytes/macrophages in the TAC mouse model. Our previous study revealed inflammatory immune cell infiltration in the TAC model, which suggested that inflammatory immune cells may be considered as potential therapeutic targets for CHF (Ni et al., 2021). We found that NXK could decrease the expression of inflammatory cytokines such as IL-1, IL-6, TNF-α, ICAM-1, and VCAM-1. Recent studies have shown that the expression of *TNF-α* and *IL-6* genes increases in CHF patients, and are positively correlated with the severity of heart failure (Schumacher and Naga Prasad, 2018), thus indicating that the expression of pro-inflammatory cytokines is a significant factor in the severity of CHF. Moreover, increased expression of ICAM-1 and VCAM-1 was observed in heart failure patients (Wrigley et al., 2013). ICAM-1 regulates cardiac inflammation and cardiac function in the TAC mouse model, suggesting that ICAM-1 is associated with pressure overload (Salvador et al., 2017).

*Ilex Pubescens* is the main component of Mao holly. Clinical trials have shown that *Ilex Pubescens* reduces the levels of TNF-α and IL-6 in patients with CHF (Zhang and Xian, 2012). Furthermore, an animal study revealed that the levels of serum IL-1-β and NF-κB were significantly decreased in rats with myocardial infarction after intervention with *Ilex Pubescens* (Zheng et al., 2014). Moreover, several studies have shown that *ginseng* exerts a strong cardioprotective effect in cardiovascular diseases *in vivo* and *ex vivo*; which is reflected in *ginseng* medicine and ginseng extracts such as ginsenosides. In the animal model of myocardial ischemia-reperfusion injury, *ginseng* protected the heart through multiple signaling pathways, such as the nitric oxide (NO) signaling pathway, which improved vascular endothelial function and reduced apoptosis by anti-oxidation (Lee et al., 2019). In this study, we found that NXK, with red *ginseng* and Mao holly as the main components, exerted anti-inflammatory properties and inhibited the expression of inflammatory factors of CHF.

TLRs are a conserved family of transmembrane protein signaling receptors that mediate innate immunity. Importantly, ventricular TLR4 contributes to adverse cardiac remodeling during chronic pressure overload (Kessler et al., 2021). TLR4 can stimulate the expression of NF-κB, which is the primary transcription factor that activates and induces inflammation in cardiomyocytes (Huang et al., 2016), thereby stimulating the expression of inflammatory cytokines, such as TNF-α and IL-6 (Boyd et al., 2006). In this study, TLR4 was decreased in the perindopril and NXK groups, which indicated that NXK and ACEI might improve the inflammatory response of CHF by dampening the TLR4/NF-κB signaling pathway. Meanwhile, the expression levels of MyD88 and P-IκB were decreased by NXK. MyD88 is a central node of inflammatory signaling pathways and the canonical adaptor for inflammatory signaling pathways downstream of the TLR family (Deguine and Barton, 2014). MyD88-independent signaling pathway can trigger an inflammatory response by activating NF-κB (Huang et al., 2016). Several studies have shown that TLR4/NF-κB signaling pathways have a key role in the inflammatory response of CVD. TLR4 accelerates the progression of atherosclerosis and increases the expression of ICAM-1 and VCAM-1, leading to the synergistic activation of NF-κB in vascular walls *in vivo* and vascular smooth muscle cells *in vitro* (Shinohara et al., 2007). NF-κB remains inactive by binding with IκB proteins under physiological conditions. Upon stimulation, the IκB kinase is phosphorylated and degraded to enhance NF-κB nuclear translocation, thereby initiating the transcription of downstream genes, including inflammatory cytokines (Deng et al., 2019). Inhibition of the TLR4/NF-κB signaling pathway decreases the expression levels of inflammatory factors and protects against the effects of ischemia and reperfusion (Zhu et al., 2018). In this study, the mRNA levels of inflammatory cytokines TNF-α, IL-1β, and IL-6 were decreased in CHF mice treated with NXK. Interestingly, the inhibition of the TLR4/NF-κB signaling pathway reduces the expression levels of inflammatory factors, which improves cardiac function and heart failure progression. Hence, NXK might decrease the inflammation level of the CHF mouse model by down-regulating the TLR4/NF-κB signaling pathway. Although our study proved that NXK could decrease the expression of TLR2 and TLR9 proteins in TAC model mice, we did not use inhibitors of TLR2/9 or gene-editing mice to confirm the specific mechanism of NXK.

An inflammatory environment promotes myocardial remodeling and myocardial fibrosis, further decreasing myocardial contractility and heart failure. In this study, the degree of myocardial fibrosis and the mRNA expression levels of α-SMA, TGF-β, collagen I, collagen III, and MMP9 were evaluated. A larger fibrotic area was seen in the myocardium of the TAC group, while there was a significantly smaller fibrotic area in the NXK group. Furthermore, the relative expression levels of α-SMA, Col1, Col3, and MMP9 were significantly decreased in the NXK middle- and high-dose groups. In addition, NXK inhibited pro-inflammatory CD11b + Ly6C + monocytes/macrophages in the TAC mouse model. A recent study reported that macrophages expressing heterogeneous immunophenotypes participate in myocardial fibrosis (Koga et al., 2021). Multiple preclinical and clinical trials have examined the inflammatory response with the overall goal of inhibiting cardiac fibrosis and progression of ischemic heart failure, with satisfactory results (Zaidi et al., 2021). Therefore, these data suggested that NXK might improve fibrosis by reducing the level of inflammation, which is a potential treatment for CHF.

However, this study had some limitations. First, the effects of *Radix ginseng Rubra* and *llex pubescens* were not evaluated. Second, inhibitors or gene-editing mice were not used to confirm the specific mechanism of TLR2 and TLR9 in NXK, which we plan to do in the future.

## Conclusion

Nuanxinkang tablet (NXK) improves heart function and reduces inflammation through the TLR-mediated NF-κB signaling pathway, and may be used as an innovative treatment strategy for CHF.

## Data Availability

The original contributions presented in the study are included in the article/Supplementary Material, further inquiries can be directed to the corresponding authors.
