# Supplementary material for: A Simplified Herbal Formula Improves Cardiac Function and Reduces Inflammation in Mice Through the TLR-Mediated NF-κB Signaling Pathway
Source: Front Pharmacol. 2022 Jun 6;13:865614. doi: 10.3389/fphar.2022.865614 (PMC9207450; doi:10.3389/fphar.2022.865614)
Supplement: Supplementary file 1 [file Table1.docx]

**Table S1.** Primer sequences used in the study

| Gene | Primer sequence (5′→3′) |
| --- | --- |
| mouse *actin* forward | GAAATCGTGCGTGACATCAAAG |
| mouse *actin* reverse | TGTAGTTTCATGGATGCCACAG |
| mouse *IL-1β* forward | TCCAGGATGAGGACATGAGCAC |
| mouse *ANP* forward | GCTTCGGGGGTAGGATTGAC |
| mouse *ANP* reverse | CACACCACAAGGGCTTAGGA |
| mouse *BNP* forward | CGGATCCGTCAGTCGTTTGG |
| mouse *BNP* reverse | AAAGAGACCCAGGCAGAGTCA |
| mouse *IL-1β* reverse | GAACGTCACACACCAGCAGGTTA |
| mouse *IL-6* forward | CCACTTCACAAGTCGGAGGCTTA |
| mouse *IL-6* reverse | GCAAGTGCATCATCGTTGTTCATAC |
| mouse *TNF-α* forward | GTTCTATGGCCCAGACCCTCAC |
| mouse *TNF-α* reverse | GGCACCACTAGTTGGTTGTCTTTG |
| mouse *VCAM-1* forward | GTTCCAGCGAGGGTCTACC |
| mouse *VCAM-1* reverse | AACTCTTGGCAAACATTAGGTGT |
| mouse *ICAM-1* forward | GTGATGCTCAGGTATCCATCCA |
| mouse *ICAM-1* reverse | CACAGTTCTCAAAGCACAGCG |
| mouse *GAPDH* forward | GGTTGTCTCCTGCGACTTCA |
| mouse *GAPDH* reverse | TGGTCCAGGGTTTCTTACTCC |
| mouse *MMP* forward | CCAGCCGACTTTTGTGGTCT |
| mouse *MMP* reverse | TGGCCTTTAGTGTCTGGCTG |
| mouse *Collagen I* forward | TGGCCTTGGAGGAAACTTTG |
| mouse *Collagen I* reverse | CTTGGAAACCTTGTGGACCAG |
| mouse *Collagen III* forward | CTGTAACATGGAAACTGGGGAAA |
| mouse *Collagen III* reverse | CCATAGCTGAACTGAAAACCACC |

**Table S2.** RNA Expression of BNP and ANP (mean (SD))

| Group | Sham | TAC | Perindopril | NXK(L) | NXK(M) | NXK(H) |
| --- | --- | --- | --- | --- | --- | --- |
| BNP  Relative Expression | 0.59  (0.07) | 2.48^***^  (0.15) | 1.80^###^  (0.14) | 0.81^###^  (0.02) | 1.69^###^  (0.17) | 1.67^###^  (0.09) |
| ANP  Relative Expression | 0.56  (0.02) | 1.82^***^  (0.14) | 1.18^###^  (0.03) | 0.76^###^  (0.08) | 0.62^###^  (0.07) | 0.43^###^  (0.10) |

Sham: sham operation group, TAC: heart failure model group, Perindopril: Perindopril group, NXK (L): Nuanxinkang tablet low-dose group, NXK (M): Nuanxinkang tablet medium-dose group, NXK (H): Nuanxinkang tablet high-dose group. Compared to the sham group *p < 0.05, **p < 0.01, ***p < 0.001, compared to the Tac group #p < 0.05, ##p < 0.01, ###p < 0.001.

**Table S3.** Myocardial fibrosis area (mean (SD))

| Group | Sham | TAC | Perindopril | NXK (L) | NXK (M) | NXK (H) |
| --- | --- | --- | --- | --- | --- | --- |
| Fibrosis area | 87.19  (6.42) | 197.78  (9.96) | 89.87  (5.05) | 95.18  (5.55) | 147.19  (10.00) | 98.51  (2.01) |
| Relative area of fibrosis (%) | 0.39  (0.08) | 0.68^***^  (0.04) | 0.37^###^  (0.04) | 0.46^###^  (0.02) | 0.51^###^  (0.04) | 0.41^###^  (0.03) |

Sham: sham operation group, TAC: heart failure model group, Perindopril: Perindopril group, NXK (L): Nuanxinkang tablet low-dose group, NXK (M): Nuanxinkang tablet medium-dose group, NXK (H): Nuanxinkang tablets high-dose group. Compared to the sham group *p < 0.05, **p < 0.01, ***p < 0.001, compared to the Tac group #p < 0.05, ##p < 0.01, ###p < 0.001.

**Table S4.** RNA Expression of α- SMA, TGF- β, Col1, Col3, MMP9 (mean (SD))

| Group | Sham | TAC | Perindopril | NXK(L) | NXK(M) | NXK(H) |
| --- | --- | --- | --- | --- | --- | --- |
| α-SMA  Relative Expression | 1.18  (0.01) | 2.33^***^  (0.08) | 1.21^###^  (0.09) | 0.82^###^  (0.11) | 0.88^###^  (0.12) | 0.93^###^  (0.14) |
| TGF-β  Relative Expression | 0.59  (0.06) | 2.48^***^  (0.12) | 1.80  (0.11) | 1.43^#^  (0.46) | 1.42^#^  (0.44) | 1.31^#^  (0.35) |
| Col1  Relative Expression | 1.10  (0.10) | 15.58^***^  (0.22) | 1.36^###^  (0.20) | / | 2.18^###^  (0.16) | 2.36^###^  (0.31) |
| Col3  Relative Expression | 1.03  (0.04) | 7.90^***^  (0.22) | 1.35^###^  (0.10) | / | 2.22^###^  (0.14) | 2.37^###^  (0.13) |
| MMP9  Relative Expression | 1.03  (0.03) | 14.28^***^  (0.10) | 2.62^###^  (0.16) | / | 3.52^###^  (0.19) | 3.77^###^  (0.21) |

Sham: sham operation group, TAC: heart failure model group, Perindopril: Perindopril group, NXK (L): Nuanxinkang tablet low-dose group, NXK (M): Nuanxinkang tablet medium-dose group, NXK (H): Nuanxinkang tablet high-dose group. Compared to the sham group *p < 0.05, **p < 0.01, ***p < 0.001, compared to the Tac group #p < 0.05, ##p < 0.01, ###p < 0.001.

**Table S5.** Expression of TLR4, MyD88 and NF-κB p65 proteins (mean (SD))

| Group | Sham | TAC | Perindopril | NXK(L) | NXK(M) | NXK(H) |
| --- | --- | --- | --- | --- | --- | --- |
| Relative expression value of TLR-4 | 0.72  (0.03) | 3.86**  (0.17) | 2.51^##^  (0.09) | 2.28^##^  (0.09) | 0.78^##^  (0.03) | 1.30^##^  (0.05) |
| Relative expression value of MyD88 | 1.00  (0.03) | 4.35***  (0.11) | 2.66^###^  (0.15) | 2.78^##^  (0.18) | 1.08^###^  (0.04) | 1.18^###^  (0.05) |
| Relative expression value of NF-κB p65 | 1.00  (0.09) | 6.67***  (0.20) | 6.65  (0.18) | 5.81^#^  (0.18) | 4.42^###^  (0.15) | 4.82^##^  (0.14) |

Sham: sham operation group, TAC: heart failure model group, Perindopril: Perindopril group, NXK (L): Nuanxinkang tablet low-dose group, NXK (M): Nuanxinkang tablet medium-dose group, NXK (H): Nuanxinkang tablet high-dose group. Compared to the sham group *p < 0.05, **p < 0.01, ***p < 0.001, compared to the Tac group #p < 0.05, ##p < 0.01, ###p < 0.001.

**Table S6** Expression of total IκBα, P-IκBα protein (mean (SD))

| Group | Sham | TAC | Perindopril | NXK(L) | NXK(M) | NXK(H) |
| --- | --- | --- | --- | --- | --- | --- |
| Total IκBα  Expression | 2.29  (0.06) | 0.51***  (0.01) | 0.88^##^  (0.03) | 0.90^###^  (0.02) | 0.94^###^  (0.02) | 0.88^##^  (0.03) |
| P-IκBα  Expression | 0.98  (0.02) | 15.46***  (0.30) | 15.15  (0.64) | 11.87^##^  (0.19) | 11.59^###^  (0.21) | 8.17^###^  (0.19) |

Sham: sham operation group, TAC: heart failure model group, Perindopril: Perindopril group, NXK (L): Nuanxinkang tablet low-dose group, NXK (M): Nuanxinkang tablet medium-dose group, NXK (H): Nuanxinkang tablet high-dose group. Compared to the sham group *p < 0.05, **p < 0.01, ***p < 0.001, compared to the Tac group #p < 0.05, ##p < 0.01, ###p < 0.001.

**Table S7** Expression of NF-κB p65, P-IκBα protein (mean (SD))

| Group | NXK(M) | LPS | LPS;  NXK(M) | TAK242 | TAK242;  NXK(M) |
| --- | --- | --- | --- | --- | --- |
| NF-κB p65 Expression | 1.56^###^  (0.05) | 2.36  (0.07) | 1.47^##^  (0.05) | 1.92^#^  (0.07) | 1.30^###^  (0.04) |
| P-IκBα  Expression | 0.95^###^  (0.06) | 1.74  (0.06) | 1.08^##^  (0.03) | 1.03^##^  (0.09) | 1.19^##^  (0.04) |

LPS: lipopolysaccharide group, TAK242:TAK242 group, NXK (M): Nuanxinkang tablet medium-dose group. Compared to the LPS group #p < 0.05, ##p < 0.01, ###p < 0.001.

**Table S8** Expression of TLR2, TLR7, TLR9 protein (mean (SD))

| Group | Sham | TAC | Perindopril | NXK(M) |
| --- | --- | --- | --- | --- |
| TLR2 Expression | 0.16  (0.03) | 1.16***  (0.05) | 0.34^##^  (0.02) | 0.51^##^  (0.02) |
| TLR7 Expression | 0.50  (0.03) | 1.23**  (0.09) | 0.68^##^  (0.05) | 1.09  (0.03) |
| TLR9 Expression | 0.55  (0.03) | 0.98***  (0.04) | 0.97  (0.07) | 0.58^##^  (0.06) |

Sham: sham operation group, TAC: heart failure model group, Perindopril: Perindopril group, NXK (M): Nuanxinkang tablet medium-dose group. Compared to the sham group *p < 0.05, **p < 0.01, ***p < 0.001, compared to the Tac group #p < 0.05, ##p < 0.01, ###p < 0.001.
